# Jan Mikulicz-Radecki (1850–1905): a fundamental contributor to world surgery; surgeon of the head, neck, and esophagus

**DOI:** 10.1007/s00405-012-1962-2

**Published:** 2012-03-16

**Authors:** Jerzy Kuczkowski, Czesław Stankiewicz, Łukasz Plichta, Joanna Cieszyńska

**Affiliations:** 1 Otolaryngology Department, Medical University of Gdańsk, Dębinki 7, 80-211 Gdańsk, Poland; 2 Otolaryngology Department, Municipal Hospital in Gdynia, Gdynia, Poland

**Keywords:** Jan Mikulicz-Radecki, Head, Neck, Esophagus surgery, Laryngology

## Abstract

We present some of many valuable and unique achievements of Jan Mikulicz-Radecki with special regard to his contribution to laryngology. He constructed esophagogastroscope, and was one of the first to perform endoscopy of esophagus and ventricle. He published several papers describing new approaches to maxillary sinus through inferior meatus, surgical management of tonsillar cancer via lateral pharyngotomy, correction of post-traumatic nasal deformations, and the use of iodophorm in healing wounds. Among Mikulicz’s many celebrated scientific achievements, the most important remains the development of asepsis and creation of a surgical school, which was a modernized continuation of Langenbeck–Billroth achievements.

Professor Jan Mikulicz-Radecki was renowned for his achievements in surgery of the digestive tract and chest, primarily due to the introduction of numerous new operative techniques across orthopedics, urology, gynecology, endocrinology, neurosurgery, and laryngology [1–4]. He belonged to a pioneering group of surgeons who developed principles of asepsis and antisepsis in operating theaters, and his innovative methods of diagnosis and treatment of the diseases of the nose, paranasal sinuses, oral cavity, throat, and esophagus achieved worldwide recognition [1, 4–9] (Fig. 1).

**Fig. 1:**
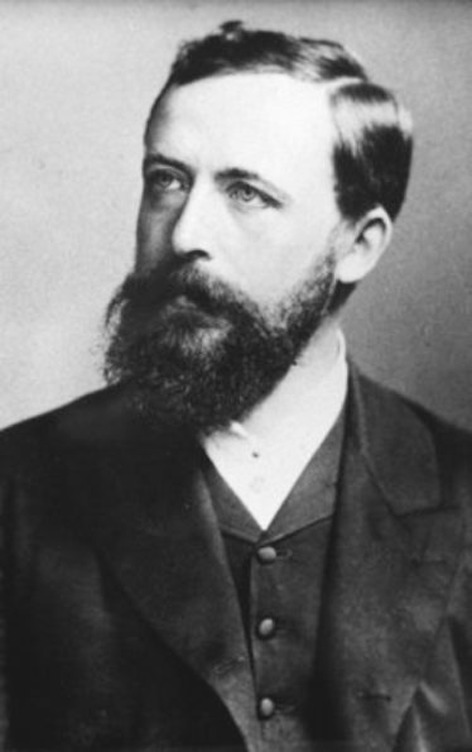
Jan Mikulicz-Radecki in Jagiellonian University in Cracow (1882–1887) (reproduced from the collection of Department of History of Medicine, Jagiellonian University in Cracow)

Mikulicz’s career may be divided into four time periods when he worked in Vienna, Cracow, Königsberg, and Breslau. During each period he introduced many unique innovations and improvements which are worth recalling. Jan Mikulicz-Radecki was born in 1850 at Czerniowice (present-day Romania); his parents were German Emilia Freiin von Damnitz and Polish Andreas Mikulicz—coat of arms Gozdawa. Between 1869 and 1875 he studied medicine at Vienna University and then commenced his professional life in the world -famous Surgical Clinic (“Allgemeine Krankenhaus”) conducted by professor Theodor Billroth. He began his scientific research with describing scleroma as an inflammatory, non-neoplastic process with unique type of cells, later called by his name [10]. Upon leaving Billroth’s Clinic, Mikulicz took over as head of the surgical department of the “Allgemeine Poliklinik” (general outpatient clinic) in Vienna. During this period he developed a technique of esophageal endoscopy. The endoscope of his design was a metal tube 65 cm long and 14 mm in diameter, bent 150 degrees between its central and lower parts. It comprised electrical wire for distal illumination (Edison’s bulb at the end of the endoscope) and pipes for filling the stomach with air and water. Using this esophagoscope Mikulicz was the first (1881) to identify cancer of the lower part of the esophagus and stenosis caused by aortic aneurysm [5, 11] (Fig. 2).

**Fig. 2:**
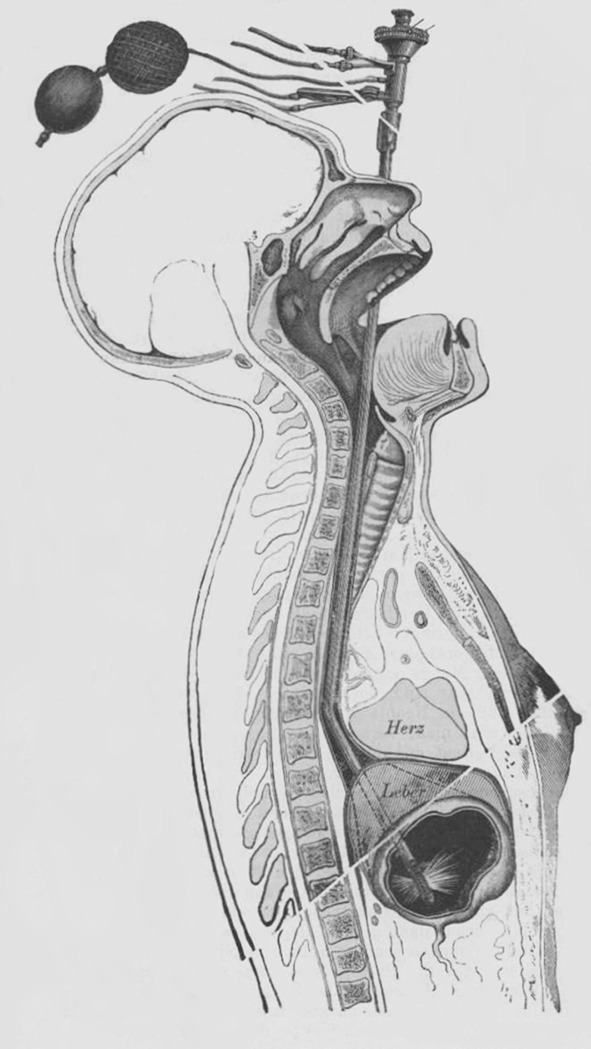
Esophageal endoscope designed by Mikulicz (reproduced from “Uber Gastroskopie und Oesophagoskopie,” Wiener medizinische Presse 1881, Bd. 22, S. 1441)

Supported by professor Billroth, Mikulicz took up the position of head of the Surgical Clinic at the Jagiellonian University of Cracow (1882–1887), where he invented a new technique of managing post-traumatic nasal deformations. He formulated the rules and divided the process into four stages. In the first phase, a surgeon should separate the skin from the cartilages and bones. The second stage involved lifting the sunken nose by means of the wire scaffolding and then fixing it with Frisch braces. In the third phase cutaneous nasal septum should be constructed from pedicled fragment of the nasal dorsal skin, while the fourth phase was nasal plasty by Susruta method [12]. In 1883, Mikulicz reported a new approach to juvenile fibroma dissection. He modified the Gussenbauer’s method and managed to access the nasal part of the pharynx through the oral cavity by choosing a longitudinal incision in the midline of the soft palate. The tumor was then removed with bent scissors. After the procedure, the wound was packed with gauze and moistened in iodophorm. Palatoplasty was performed within 3 weeks after the tumor’s removal. This method caused relatively little deformation to the oral cavity and no deformation to the patient’s face with little blood loss during operation [13]. One of many Mikulicz’s achievements was inventing a new surgical approach to the treatment of tonsillar cancer via lateral pharyngotomy. Broad access to the tonsillar tumors and base of the tongue was achieved via mandibulotomy, with a marginal bundle of the facial nerve as well as masseter and medial pterygoid muscles preservation. The advantage of this procedure was opening the lateral pharyngeal wall leaving the oral cavity not communicated with the wound [6, 8, 14]. In 1886 Mikulicz presented eight cases of the tonsillar cancer with well-described symptomatology and reported another new technique of accessing maxillary sinus through the inferior nasal meatus in case of an empyema [7–9].

During his stay at the University of Königsberg (1887–1890), Mikulicz introduced a steam boiler for sterilization of the operative tools. In 1888, at the meeting of the Scientific Medical Society in Königsberg, he presented for the first time a case of bilateral nodular edema of the lachrymal and salivary glands, later called the Mikulicz’s syndrome [15] (Fig. 3).

**Fig. 3:**
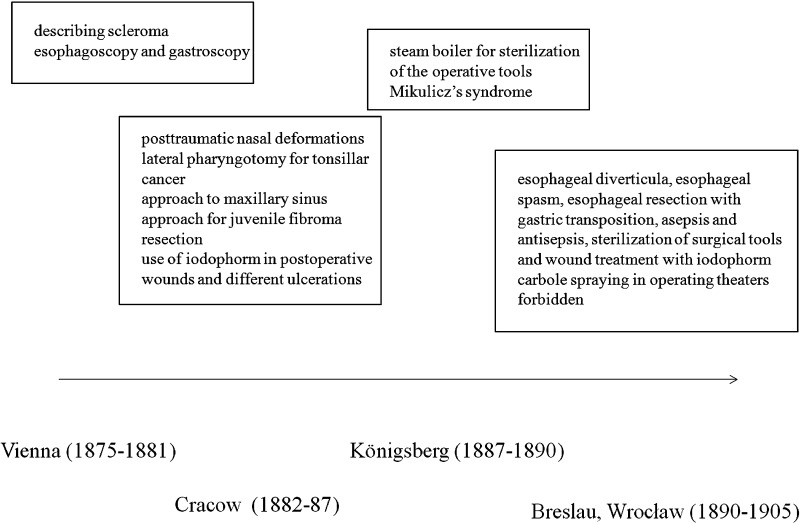
Time table of professor Mikulicz-Radecki’s achievements

The most significant scientific and professional achievements, however, pertain to the period when Mikulicz was head of the Surgical Clinic at the University of Breslau (1890–1905). The newly designed and perfectly equipped Clinic consisted of 90 beds and an operating theater, one of the most modern in Europe. During this period Mikulicz spent most of his time operating; furthermore, he published several papers on the treatment of esophageal diverticula, esophageal spasm, esophageal resection with gastric transposition, asepsis and antisepsis, sterilization of surgical tools, and wound treatment with iodophorm. He introduced an ointment with 1% lapis solution with addition of Peruvian balsam for chronic ulcers. He then started to propagate a rule known as *dictum Mikuliczi*, which stated: “administration of narcosis in a patient, whose hemoglobin dropped below 30%, is extremely dangerous” [1, 15]. Mikulicz invented several surgical tools, which are used until now (e.g., harpoon trocar, forceps to grasp the omentum). Together with von Bruns he proved that the influence of contaminated air on wound infection is relatively insignificant, and therefore forbade carbole spraying in operating theaters, which as they claimed, was equally poisonous for the patients as for bacteria. Mikulicz was not only a great doctor with strong and immaculate character, but also a talented surgeon, who operated quickly and firmly, without significant blood loss. He believed that surgical efficacy is based on the right diagnosis and always acted according to the rules: “salus aegroti suprema lex” (The well-being of the patient is the most important law) and “primum non nocere” (*First, do no harm*). In recognition of his merits, the German Surgical Society (*Deutsche Gesellschaft für Chirurgie*) every year grants an award in surgery for the best paper in the field of endoscopy and laparoscopy “Von Mikulicz–Kelling-Preis” [1, 4].
